# Responses of soil microbial biomass and enzyme activity to herbicides imazethapyr and flumioxazin

**DOI:** 10.1038/s41598-020-64648-3

**Published:** 2020-05-06

**Authors:** Mariane Pertile, Jadson Emanuel Lopes Antunes, Fabio Fernando Araujo, Lucas William Mendes, Paul J. Van den Brink, Ademir Sérgio Ferreira Araujo

**Affiliations:** 1 Soil Quality Lab., Agricultural Science Center, Federal University of Piauí, Teresina, PI Brazil; 2 University of West São Paulo, UNOESTE, Presidente Prudente, SP, Brazil; 3 Laboratory of Molecular Ecology, CENA-USP, Piracicaba, SP, Brazil; 4 Wageningen Environmental Research, Wageningen, The Netherlands; 5 Aquatic Ecology and Water Quality Management Group, Wageningen University, Wageningen, The Netherlands

**Keywords:** Microbiology, Environmental sciences

## Abstract

The use of herbicides is important for controlling weeds in crops. However, they can present impacts on soil properties, such as biological properties. In this study, we evaluated the responses of soil microbial biomass and enzymes activity to the application of the herbicides imazethapyr and flumioxazin and their mixture in an experiment under laboratory conditions, using soils with a different history of use. Soil microbial biomass C (MBC) decreased, while microbial biomass N (MBN) was not affected after the application of the herbicides as compared to the control. Soil respiration, respiratory quotient, and dehydrogenase (DHA) activity increased significantly after the application of the herbicides compared to the control. The hydrolysis of fluorescein diacetate (FDA) was not significantly different between the control and the herbicide treatments. The principal response curve showed the largest initial effects for the flumioxazin, followed by imazethapyr and their mixture. Flumioxazin had a different influence on soil respiration and respiratory quotient than imazethapyr and their mixture. Finally, the effects of herbicides on soil microbial biomass and enzymes are short-term as we observed recovery in the biological parameters over time.

## Introduction

The use of herbicides, as an effective practice for controlling weeds in crops, has increased in the agricultural systems in Brazil mainly due to the introduction of herbicide-resistant plants in agriculture, such as soybean and maize^1^. In Brazil, herbicides represent about 60% of the total pesticides used in agriculture^2^. Although herbicides are important for agriculture, there is a concern about their fate in the environment and their impact on soil biological properties^3^.

Biological properties are critically important to the ecosystem functioning since they are involved in soil organic matter decomposition, nutrient cycling, and degradation of pesticides, such as herbicides^4^. Therefore, studies assessing the effect of herbicides on soil biological properties are important for evaluating soil quality and health^5^. In addition, soil biological properties are more effective as indicators of soil quality than physical and chemical properties as they often show a faster response to an environmental impact^6^.

As important and responsive biological properties, soil microbial biomass and enzyme activities are frequently recommended for evaluating the effects of herbicides on the soil environment^7^. Soil microbial biomass represents the active part of soil organic matter and is involved in several functions in soil, presenting a rapid turnover of soil C, N, and P; while enzymes are a suitable indicator of the catabolic activity of soil microorganism^6^. These biological properties are highly sensitive to detect soil disturbance after the application of chemicals, such as herbicides^4,7,8^. For example, glyphosate, one of the most important herbicides used is soybean crops, presents a transitory and short-term effect on soil microbial biomass and activity^9^.

Currently, glyphosate is being replaced by imazethapyr and flumioxazin in the weeds control in soybean crops, since these herbicides provide a high spectrum of action against weeds^10^. Imazethapyr, an herbicide belonging to the imidazoline family, acts on the grass and broadleaf weeds, being recommended for use in soybean cultivation^11^. It has a mode of action on cell metabolism and could influence the accumulation of microbial C^12^. Flumioxazin, that belongs to the N-phenylphthalimide chemical family, is a soil-applied herbicide recommended for broadleaf weeds control in soybean, peanut, and vineyard^13^. It has a mode of action on the protoporphyrinogen oxidase^12^, presenting anti-microbial effect and could inhibit some enzymes^14^.

The application of imazethapyr in the soil has shown a different effect on soil microbes. A previous study conducted by Perucci and Scarponi^15^ has shown that imazethapyr, when applied at the recommended field rate for soybean (1.6 mg kg^−1^), had no adverse effect on soil microbial biomass and activity. However, in a field study during two years, Zhang et al^16^. have found that the application of imazethapyr (0.1, 1 or 10 mg kg^−1^ soil) changed the content of microbial biomass C. These studies have shown that would be an influence of the history of application on soil microbial biomass and, probably, enzymes activity. On the other hand, there are no studies about the effect of flumioxazin on soil microbial biomass and enzyme activity. So far, the available studies about flumioxazin focused on its dissipation and movement in the soil, rather than its effects on microbial biomass^13,17^.

Although the use of a mixture of herbicides seems to be more effective in controlling weeds^10^, it could present a complex and larger effect on non-target organisms than the individual compounds^18^. Therefore, studies evaluating the effects of the separate application of the herbicides or in a mixture on soil microbial biomass and enzyme activity are necessary for a better understanding of their effects on soil biological properties. In this context, we hypothesized that (1) the history of herbicides application and their different mode of actions could influence the soil microbial biomass and enzyme activity; and (2) there would be a different effect of the mixture in comparison with the individual compounds. We, therefore, addressed the responses of soil microbial biomass and enzymes activity to the application of imazethapyr and flumioxazin and their mixture in a tropical soil.

## Results

**Figure 1:**
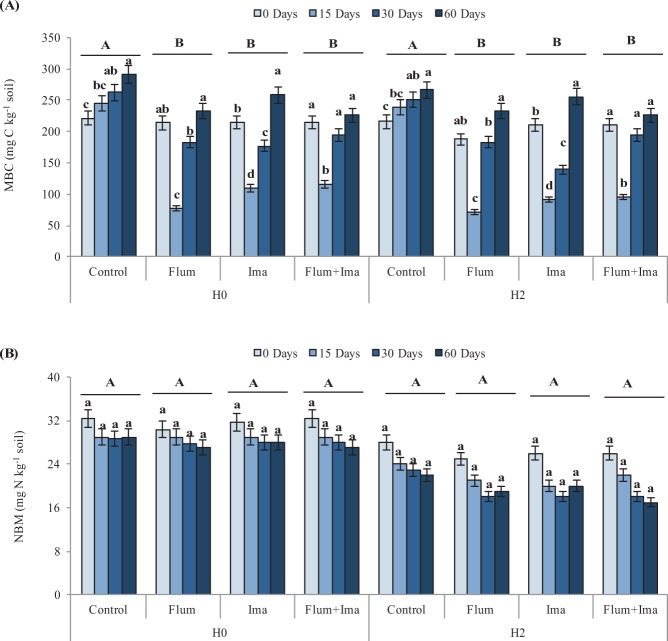
Microbial biomass C (**A**) and N (**B**) in soils, without (H0) and with (H2) history of herbicides application in the field, untreated (control) and treated with Flumioxazin (Flum), imazethapyr (Ima) and their mixture (Flum+Ima), at different incubation times. Bars represent the SD of the mean. The different lower-case letters above the bars indicate significant differences (P < 0.05) between sampling times for each treatment and different upper-case letters above the bars indicate significant differences (P < 0.05) between treatments (mean values) for each soil.

**Figure 2:**
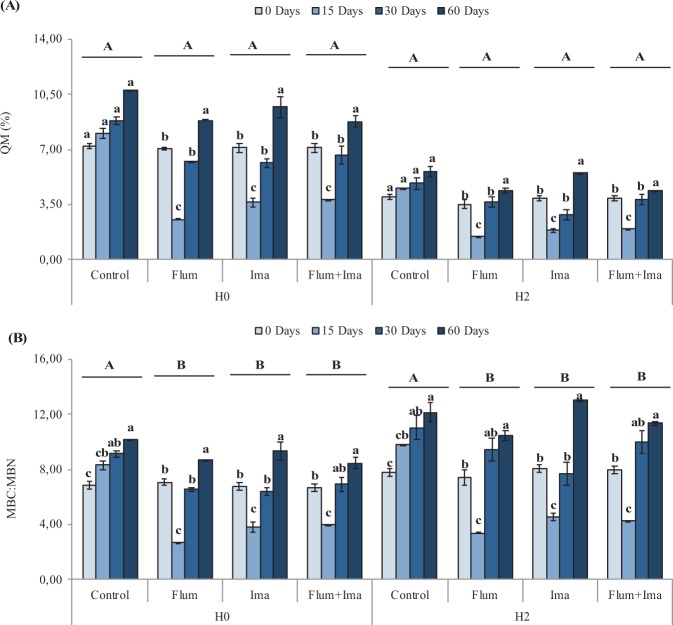
Microbial quotient (QM) (**A**) and MBC:MBN ratio (**B**) in soils, without (H0) and with (H2) history of herbicides application in the field, untreated (control) and treated with Flumioxazin (Flum), imazethapyr (Ima) and their mixture (Flum + Ima), at different incubation times. Bars represent the SD of the mean. The different lower-case letters above the bars indicate significant differences (P < 0.05) between sampling times for each treatment and different upper-case letters above the bars indicate significant differences (P < 0.05) between treatments (mean values) for each soil.

Soil microbial biomass C (MBC) decreased significantly after the application of the herbicides as compared to the control in both areas with (H2) and without (H0) a previous application of the herbicides (Fig. 1A), while microbial biomass N (MBN) was not significantly different between the control and the herbicide treatments (Fig. 1B). During the incubation, MBC decreased at 15 days after herbicides application and increased at 30 and 60 days. In contrast, MBC increased from 0 to 60 days in the control. On the other hand, MBN did not decrease significantly from 0 to 60 days. The microbial quotient (QM) did not show differences between the control and the herbicides treatments (Fig. 2A), while MBC:MBN ratio decreased after the application of the herbicides as compared to the control in both H0 and H2 (Fig. 2B). During the incubation, QM, and MBC:MBN ratio decreased at 15 days after herbicides application and increased at 30 and 60 days (Fig. 2).

**Figure 3:**
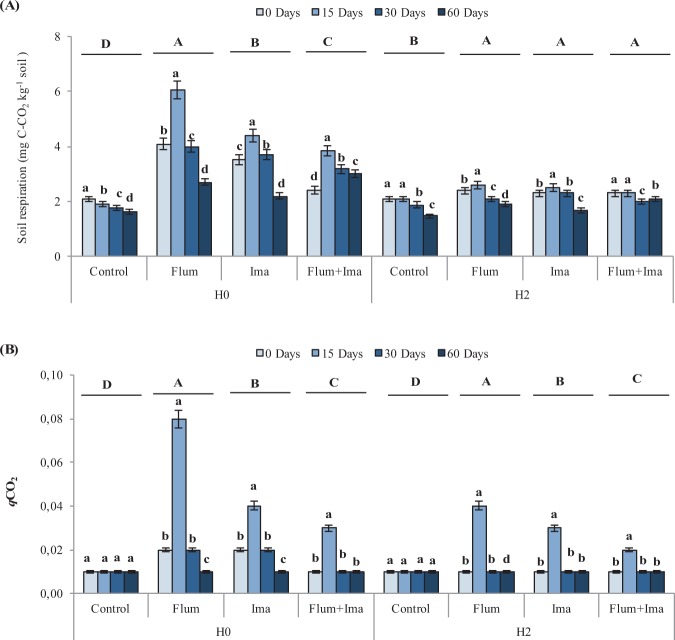
Soil respiration (**A**) and respiratory quotient (**B**) in soils, without (H0) and with (H2) history of herbicides application in the field, untreated (control) and treated with Flumioxazin (Flum), imazethapyr (Ima) and their mixture (Flum+Ima), at different incubation times. Bars represent the SD of the mean. The different lower-case letters above the bars indicate significant differences (P < 0.05) between sampling times for each treatment and different upper-case letters above the bars indicate significant differences (P < 0.05) between treatments (mean values) for each soil.

Soil respiration (Fig. 3A) and respiratory quotient (Fig. 3B) increased significantly after the application of the herbicides as compared to the control in both H0 and H2 soils. During the incubation, soil respiration and respiratory quotient increased, in both H0 and H2, at 15 days after herbicides application and decreased at 30 and 60 days. Interestingly, soil respiration decreases and respiratory quotient did not vary during the period of incubation in the control (Fig. 3).

**Figure 4:**
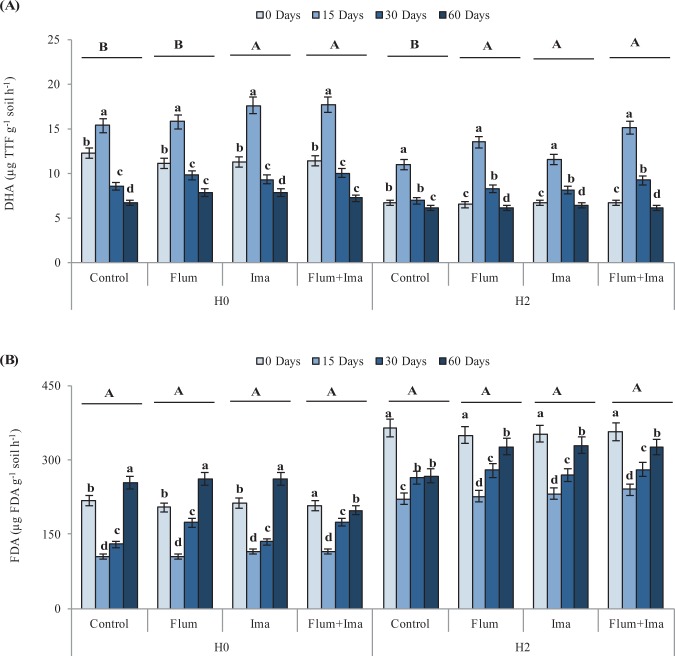
Dehydrogenase activity (**A**) and fluorescein diacetate hydrolysis (**B**) in soils, without (H0) and with (H2) history of herbicides application in the field, untreated (control) and treated with Flumioxazin (Flum), imazethapyr (Ima) and their mixture (Flum+Ima), at different incubation times. Bars represent the SD of the mean. The different lower-case letters above the bars indicate significant differences (P < 0.05) between sampling times for each treatment and different upper-case letters above the bars indicate significant differences (P < 0.05) between treatments (mean values) for each soil.

Except for flumioxazin in H0 soil, dehydrogenase activity (DHA) increased significantly after the application of the herbicides as compared to the control in both H0 and H2 soils (Fig. 4A), while the hydrolysis of fluorescein diacetate (FDA) was not significantly different between the control and the herbicide treatments (Fig. 4B). During the incubation, DHA increased in both H0 and H2 at 15 days after herbicides application and decreased at 30 and 60 days. In contrast, FDA decreased in both H0 and H2 at 15 days after herbicides application and increased at 30 and 60 days.

**Figure 5:**
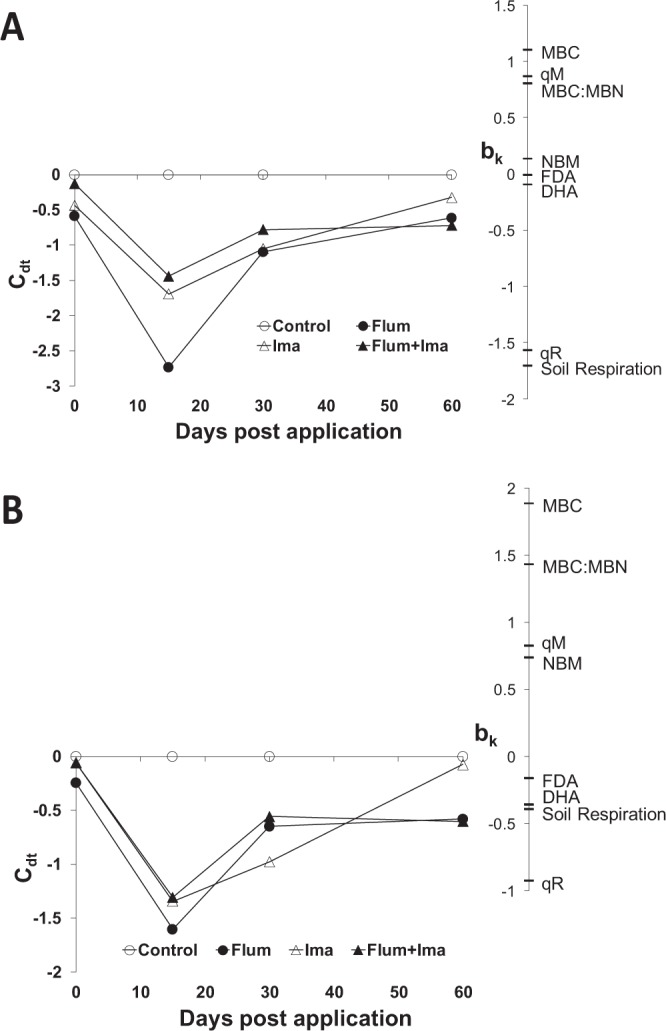
PRC diagrams showing the response of the biological parameters to the herbicide treatments for H0 (**A**) and H2 (**B**) during the incubation time. Incubation time explained 58 and 67% of the total variation in the biological parameter values of the H0 and H2 data sets, respectively. Treatment explained 41 and 31% of the H0 and H2 data sets, of which 88 and 87% are displayed in the respective PRC diagrams.

The Principal Response Curves (PRC) analysis showed that a significant part of the treatment variation was displayed in both H0 and H2 PRC diagrams (Fig. 5). The H0 soil samples showed the largest initial effects for the flumioxazin, followed by imazethapyr and their mixture (Fig. 5A). Partial recovery was also indicated. MBC, QM, and the MBC:MBN ratio decreased in values due to the treatments, while the respiratory quotient and soil respiration increased. MBN, DHA, and FDA showed no response. The H2 soil samples showed equal effect sizes for the three treatments and a full recovery for the imazethapyr and partial recovery for the flumioxazin and their mixture (Fig. 5B). MBC, MBN, QM, and the MBC:MBN ratio decreased in values; respiratory quotient, soil respiration, and DHA increased while FDA showed no response. For both the H0 and H2 soils, the treatments had a significant effect on the biological parameters at all periods of incubation. The individual treatments could not be tested against the control due to a limited number of permutation possibilities.

## Discussion

In this study, we assessed the effect of the herbicides imazethapyr, flumioxazin, and their mixture on soil microbial biomass and enzyme activity in soil with (H2) and without (H0) previous application of these herbicides in the field. Partially, in contrast with the first hypothesis, we did not find the influence of the previous application of herbicides on soil microbial biomass and respiration (Figs. 1 and 3). However, the enzyme activity was influenced by the previous application of herbicides (Fig. 4).

According to Moorman^19^, the cumulative effect of repeated annual applications of herbicides may, in some cases, not influence soil biological properties and it may be related to degradation time (DT) of the compound in the soil. Although in this study we did not evaluate DT of these herbicides in the soil, the available data have reported that the DTs of imazethapyr and flumioxazin, in similar soil types, ranges from 7–19 and 15–20 days, respectively^20^. Therefore, in our soils, both herbicides seem to be non-persistent and would not have an influence on the responses of soil biological parameters after two years.

MBC was influenced by herbicides application in both H0 and H2 as compared to the control, while MBN was not influenced by the herbicides (Fig. 1). Interestingly, MBC decreased due to the herbicides applications while MBN was not affected. It suggests that these herbicides do not have a toxic effect on microbial N, which agrees with Sawicka and Selwet^18^, who reported that imazethapyr applied at 90 g ha^−1^ does not present a negative effect on N related microorganisms. In this study, we have applied imazethapyr at 106 g ha^−1^, which is closer to the rate used by Sawicka and Selwet^21^. For flumioxazin, there is no study about this herbicide on soil microbial biomass N.

During the incubation, MBC decreased in the first 15 days after herbicides application (Fig. 1A). It agrees with Zhang et al^16^. who observed that MBC decreased during the initial incubation period after application of imazethapyr at 0.1, 1 and 10 mg kg^−1^ soil. There are no previous studies on the effect of flumioxazin on MBC and, therefore, our results suggest that flumioxazin, initially, had a negative effect on soil MBC. The results also show that after the initial negative effect, MBC recovered and increased until the end of the incubation period. This recovery of the soil microbial biomass C may be related to the degradation of these herbicides and their decreasing toxicity for soil microbial biomass^15^. Also, the initial lyses of microbial cells promoted by herbicides could have increased the content of C and, thus, contribute to C and energy sources for soil microbial biomass.

The MBC:MBN ratio was influenced by herbicides application in both H0 and H2 as compared to the control, while the microbial quotient (QM) was not influenced by the herbicides (Fig. 2). During the incubation, both parameters decreased during the first 15 days after the herbicide application and recovered to control values after this initial period. This response is similar to the one observed for MBC and indicates that any effect of herbicides on microbial biomass C influences the status of these indices.

Soil respiration is considered an indicator of microbial activity^22^, while respiratory quotient is a useful indicator of ecological disorder or disturbance in soil^23^. The results showed that soil respiration and respiratory quotient increased after the application of herbicides in both H0 and H2 as compared to the control (Fig. 3). Although the increase in soil respiration could suggest a positive effect of the herbicides on microbial activity, the increase in respiratory quotient indicates an initial negative effect of the herbicides on soil microorganisms. During the incubation, soil respiration and respiratory quotient increased during the first 15 days. It occurs since the application of chemical compounds in soil requires an adaptation of soil microbial biomass that uses their reserves to degrade these compounds. Thus, C from microbial biomass is lost, increasing the respiratory quotient. After 15 days, these parameters decreased back to control levels and this pattern could suggest an adaptation of the remaining microbial biomass that increased between 15 to 60 days.

DHA did not vary between the treatments and the control with the application of flumioxazin in the H0 soil, suggesting no effect of flumioxazin on catabolic activity in soil^24^. However, DHA was affected by herbicides in H2 soil (Fig. 4A). After the incubation, DHA increased during the first 15 days and decreased after that period to control levels. This pattern was similar to the one observed for soil respiration (Fig. 3A) and it can indicate an initial stimulation of microbial activity by the herbicides. Interestingly, the FDA was not influenced by herbicides application in both H0 and H2 as compared to the control (Fig. 4B) suggesting no detrimental effect on soil microbial activity.

Finally, we show the pattern of these biological properties in response to herbicides using the multivariate method of PRC. PRC showed significant positive and negative effects of herbicides on soil biological parameters (Fig. 5). Interestingly, the herbicide flumioxazin affected negatively the MBC and their correlated parameters, i.e. microbial quotient and MBC:MBN ratio, in the soil with no history of herbicide application (Figs. 1A, 2 and 5A). It suggests that the C-related microorganisms did not adapt to this herbicide even being applied for two years. In contrast, the parameter related to soil disturbance, i.e. respiratory quotient, increased as a response to the possible stress caused by the herbicides to soil microbial biomass (Figs. 3B and 5). When we consider the soil with previous application of the herbicides, separated and in a mixture, they present the same effect on biological parameters with negative and positive responses to microbial biomass and respiration, respectively (Fig. 5B). However, the biological parameters present a full recovery in the treatment with the application of imazethapyr, which suggests that the microbial biomass can be more adapted to this herbicide than to flumioxazin. Previously, Perucci and Scarponi^15^ have also found a recovery of microbial properties after the application of imazethapyr when it was applied at the recommended field rate.

### Ecological implications

The results of this study show that the evaluated parameters increased, decreased or did not show response during the first 15 days to the application of herbicides. MBC, QM, MBC:MBN ratio and FDA decreased initially after the application of herbicides. On the other hand, soil respiration, respiratory quotient, and DHA increased as a direct effect of herbicides. These responses can be related to the mode of action of the herbicides. The herbicide imazethapyr belongs to the class of imidazolinones and has a mode of action on cell metabolism, inhibiting acetohydroxyacid synthase (AHAS) and regulating the biosynthesis of some compounds^25^. Thiour-Mauprivez *et al*.^12^. reported AHAS influencing on the accumulation of microbial C and the microbial activity, such as FDA hydrolysis. Also, since AHAS disrupts the accumulation of microbial C, this element can be lost through catabolic respiration, i.e. increasing respiratory quotient and dehydrogenase.

Regarding flumioxazin, it belongs to the N-phenylphthalimide chemical family and has a protoporphyrinogen oxidase mode of action^12^. So far, no ecotoxicological studies were done with flumioxazin. Although N-phenylphthalimide chemicals have an anti-microbial effect and inhibit some enzymes^14^, this compound can protect N from its catabolisms^26^. Thus, although flumioxazin decreases temporally the microbial biomass C, it may have protected microbial biomass N, so presenting a neutral effect (Fig. 1).

The responses of soil biological parameters to herbicides can indicate some ecological implications for soil quality. The application of herbicides could decrease temporally the C pool, microbial stoichiometry, and activity. This result indicates a C limitation in soil and also a decrease in the organic matter decomposition by microbes^27^. On the other hand, herbicides promoted losses of C, by catabolic respiration, as an indication of microbial disturbance^23^. Interestingly, these herbicides did not present an effect on N storage in soil and potential N cycling^28^. Finally, both herbicides are degraded rapidly in the soil through microbial degradation. Thus, it could explain this temporary effect of these herbicides on soil biological parameters.

## Conclusions

In this study, the application of herbicides influenced the soil microbial biomass and enzyme activity differently. In general, the application of the herbicides influenced the soil microbial biomass C, while the hydrolysis of the FDA was not affected. Flumioxazin had a different influence on soil respiration and respiratory quotient than imazethapyr and their mixture. The mixture did not present different effects on soil microbial biomass and enzyme activity than the individual compounds. Finally, the effects of herbicides on soil microbial biomass and enzymes are short-term as we observed a recovery in the biological parameters over time.

## Material and methods

### Soil samples

Soybean fields from Iria Farm, located at Sambaiba city, Maranhao, Brazil (7°31'59“S and 46°2′6″W, 243 m), which present areas with and without a previous application of the herbicides imazethapyr, flumioxazin and their mixture, were selected for soil sampling. In the areas with previous applications of herbicides, Imazethapyr Plus NORTOX (imazethapyr), Flumyzin 500 (flumioxazin) and Zethamaxx (imazethapyr + flumioxazin) were applied, separately, during two years. These herbicides were applied in their recommended field rates which corresponded to the application of 106 g of imazethapyr ha^−1^ (1 L Imazethapyr Plus NORTOX ha^−1^ with a purity of 106 g a.i. L^−1^), 20 g ha^−1^ of flumioxazin (40 g Flumyzin 500 ha^−1^ with a purity of 500 g a.i. kg^−1^) and a mixture of 127 g ha^−1^ imazethapyr and 60 g ha^−1^ flumioxazin (0.6 L Zethamaxx ha^−1^ with a purity of 212 g a.i. L^−1^ imazethapyr and 100 g a.i. L^−1^ flumioxazin).

Soil samples were collected at areas with and without a previous application of the herbicides, from the surface layer of the soil up to a depth of 20 cm and were passed through a 2-mm sieve to remove large residue fragments. Soil pH, exchangeable Ca^2+^, Mg^2+^, K^+^, and the available P were estimated according to EMBRAPA^29^. Total organic C (TOC) was determined by wet combustion using a mixture of 5 mL of 0.167 mol L^−1^ potassium dichromate and 7.5 mL of concentrated sulfuric acid under heating (170 °C for 30 min)^30^. The chemical properties of the soils are shown in Table 1.

**Table 1:** Chemical properties of the soils used in this study.

Soil	pH	Al^3+^	Ca^2+^	Mg^2+^	K^+^	P	TOC
	CaCl_2_	cmol_c_ kg^-1^	cmol_c_ kg^-1^	cmol_c_ kg^-1^	cmol_c_ kg^-1^	mg kg^-1^	g kg^-1^
H0	5.0	0.8	0.92	0.66	0.3	2.83	30.72
H2	4.2	0.7	1.78	0.57	0.2	4.04	53.81

H0 – soil without history of herbicides application; H2 - soil with 2 years of herbicides application; TOC – total organic C.

### Incubation experiment

The soil samples collected at the areas with (H2) and without (H0) a previous application of the herbicides were used for the incubation experiment with the herbicides. The experiment had a completely randomized design with three replications that had the following treatments: imazethapyr (Ima); flumioxazin (Flu); flumioxazin + imazethapyr (Flu + Ima); and control without herbicide application.

The soil samples were treated, at the laboratory, with herbicides imazethapyr (Ima), flumioxazin (Flu) and their mixture (Flu + Ima). These herbicides were applied at the recommended field rates. Each soil sub-sample (1 kg; dry weight) received, respectively, 0.8 mg of imazethapyr (7.5 µL of Imazethapyr Plus NORTOX with a purity of 106 g a.i. L^−1^), 0.3 mg of flumioxazin (0.6 mg of Flumyzin 500 with a purity of 500 g a.i. kg^−1^), and a mixture of 0.8 mg imazethapyr and 0.38 mg flumioxazin (3.75 µL of Zethamaxx with a purity of 212 g a.i. L^−1^ imazethapyr + 100 g a.i. L^−1^ flumioxazin) that were diluted in 100 mL of water and sprayed and mixed to the soils. The rates of herbicides per kg of soil were calculated considering the mass of soil in a 0–20 cm layer of a hectare. As a control, 100 mL water was sprayed and mixed soil sub-samples.

Soil moisture content was adjusted to two-thirds of the field capacity and it was controlled every week through the gravimetric method. Each soil sub-samples were incubated in pots (1 kg; three replicates by treatments) in the dark for 60 days at 25 °C. Sub-samples of soil were removed from each pot for biological analysis at 0, 15, 30 and 60 days. The analysis of day 0 means that the biological data were collected immediately after the herbicide application.

### Biological parameters

The soil respiration was monitored during aerobic incubation procedure over seven days by measuring the CO_2_ evolved from 50 g of soil^22^. MBC and MBN were determined in 20 g soil by the chloroform fumigation-extraction method according to Vance *et al*.^31^, and Brookes et al^32^., respectively. The extraction efficiency coefficients of 0.38 and 0.45 were used to convert the difference in C and N between fumigated and unfumigated soil in MBC and MBN, respectively. Moreover, we calculated the QM, as the ratio between MBC and TOC (expressed in %), and also the ratio between MBC and MBN. The respiratory quotient was calculated as CO_2_-C unit^−1^ microbial biomass C day^−1^. Two grams of soil were used for estimating FDA hydrolysis according to the method of Schnurer and Rosswall^33^. DHA activity was determined using the method described in Casida *et al*.^34^. and based on the spectrophotometric determination of triphenyl tetrazolium formazan (TTF) released by 5 g of soil during 24 h at 35 °C. The data were collected at 15, 30 and 60 days. All biological analyses were conducted in triplicate and expressed as dry weight. The data were compared between treatments through the analysis of variance by ANOVA followed by post-hoc Newman-Keuls test. The means were compared by using the least significant difference values calculated at a 5% level.

### Principal responses curves

The biological data were analyzed using the PRC method^35^. PRC is a multivariate analysis method based on the ordination technique developed for the analysis of multivariate data sets, describing the response of communities or a set of response variables to stress in time, using a non-stressed control as a reference^35^. In order to make all parameters mathematically equally important in the analysis, they were standardized to zero mean and unit variance before analysis^35^. A separate PRC was performed for each history of previous herbicides application in field (0 and 2 years), while the statistical significance of the effects of the treatments was tested against the control for each incubation period using Monte Carlo permutation tests.
